# Scutellarein Protects Against UVB-Induced Skin Injury in a Mouse Model

**DOI:** 10.3390/molecules30193867

**Published:** 2025-09-24

**Authors:** Yue Sun, Pengfei Zhang, Fang Yang, Wang Zhang, Gaofu Li, Lei Zhou, Tiantian Xia, Yue Gao, Wei Zhou

**Affiliations:** 1 School of Pharmacy, Qinghai University, Xining 810016, China; sunyuee2022@163.com (Y.S.); fangyang@qhu.edu.cn (F.Y.); zhangwangqhu831x@163.com (W.Z.); 2 Beijing Institute of Radiation Medicine, Beijing 100850, China; sdyxyzpf@163.com (P.Z.); ligaofu543@163.com (G.L.); zhoulei970322@163.com (L.Z.); 3 Department of Tranditional Chinese Medicine, Qinghai Unversity Medical College, Xining 810016, China; xiatianttx@163.com

**Keywords:** ultraviolet damage, inflammation, oxidative stress, scutellarin, skin protection

## Abstract

UVB radiation penetrates the epidermis and upper dermis, compromising skin barrier function. This activates pro-inflammatory cells, releasing mediators (e.g., histamine, interleukins) that induce edema. UVB also generates excessive reactive oxygen species (ROS), causing oxidative stress in skin cells. Although the mechanisms of UV-induced skin damage have been extensively studied, the development of effective UV-protective drugs remains a significant challenge. Scutellarin, a flavonoid glycoside predominantly isolated from Erigeron breviscapus, has demonstrated diverse bioactivities including anti-inflammatory, antioxidant, and anti-tumor effects. However, its role in UVB-induced skin damage has not been fully explored. Therefore, we established a UVB-induced skin damage model in mice by irradiating the dorsal skin with a dose of 300 mJ/cm^2^ UVB. Through measurements of transepidermal water loss, detection of barrier-related proteins, assessment of inflammatory factors, and evaluation of oxidative stress indicators, we found that scutellarin can maintain barrier integrity, reduce skin edema, suppress inflammatory responses, and decrease oxidative stress. Moreover, RNA sequencing of mice skin revealed that scutellarin can modulate inflammatory responses and maintain extracellular matrix homeostasis to alleviate skin damage. These findings suggest that scutellarin is a natural compound with potential for UV-protective effects on the skin.

## 1. Introduction

As the largest interface organ of the human body, the skin establishes a dynamic protective barrier through its stratified structure [1]. This barrier not only participates in the regulation of systemic homeostasis but also shields against external stimuli such as Ultraviolet B [2]. Ultraviolet B (UVB) radiation elicits multifaceted damage to human skin. UVB radiation directly damages cellular DNA within skin cells, primarily through the formation of photoadducts such as pyrimidine dimers [3]. This DNA damage impairs cellular function and promotes mutagenesis. Concurrently, UVB exposure potently exacerbates inflammatory responses, characterized by the activation of immune cells and the release of inflammatory mediators, including cytokines and chemokines [4,5]. Critically, these mediators not only induce localized erythema, edema, and pain but also amplify and perpetuate the damaging effects of UVB on the skin. Concurrently, UVB irradiation generates reactive oxygen species (ROS) that surpass the scavenging capacity of the skin’s endogenous antioxidant defense system. The resultant ROS excess targets cellular constituents, including membrane lipids, proteins, and DNA, inducing oxidative damage and cellular dysfunction [6]. This oxidative stress not only accelerates the skin aging process but furthermore elevates the risk of skin carcinogenesis.

Although numerous agents for preventing and treating UVB-induced damage are currently available on the market, these pharmacological interventions are often associated with inherent limitations and potential side effects. Corticosteroids can rapidly mitigate inflammation and erythema; chronic administration may induce adverse effects such as skin thinning, telangiectasia, and hyperpigmentation. Moreover, this is frequently accompanied by a significant rebound phenomenon upon discontinuation [7,8]. Among retinoids, tretinoin effectively treats photoaged skin, improving texture and reducing wrinkles. However, initial treatment often causes noticeable adverse reactions such as erythema, burning, scaling, and dryness. It also increases skin sensitivity to ultraviolet radiation [9]. In contrast, inflammatory skin conditions such as psoriasis or contact dermatitis may be exacerbated or triggered by ultraviolet radiation exposure. Statins have been investigated due to their anti-inflammatory, immunomodulatory, and antioxidant properties. Although promising, the clinical utility of statins is curtailed by their own risk of significant cutaneous adverse effects, including eczema, photosensitivity reactions, and even necrosis [10].

Scutellarin, a flavonoid natural compound derived from Erigeron breviscapus, is a traditional Chinese medicinal agent. It is traditionally recognized for alleviating cold symptoms, relieving rheumatic pain or arthritis, activating blood circulation, and exerting anti-inflammatory and analgesic effects [11]. Contemporary research indicates that scutellarin demonstrates significant therapeutic efficacy in diverse pathological contexts, including anti-tumor activity, anti-fibrotic effects, and obesity management [12,13,14]. Although scutellarin has been well-documented to possess potent anti-inflammatory and antioxidant pharmacological activities, its protective and reparative effects on cutaneous damage remain unexplored. Therefore, this study aims to investigate the protective effects of scutellarin against UV-induced skin damage using an ultraviolet irradiation-induced mouse skin injury model. Using genomics-wide sequencing technology, we further seek to elucidate its underlying mechanisms of action, thereby establishing a comprehensive scientific foundation for its potential therapeutic applications in managing UV-induced cutaneous damage. However, there are significant differences in skin structure between mice and humans, such as epidermal thickness, pigmentation, and overall response to UVB exposure, which may lead to variations in the efficacy of scutellarein between species. Future studies should focus on bridging these species-specific gaps to further confirm the therapeutic potential of scutellarin in human applications.

## 2. Results

### 2.1. UVB Irradiation Leads to Progressive Alterations in Skin Appearance

**Figure 1:**
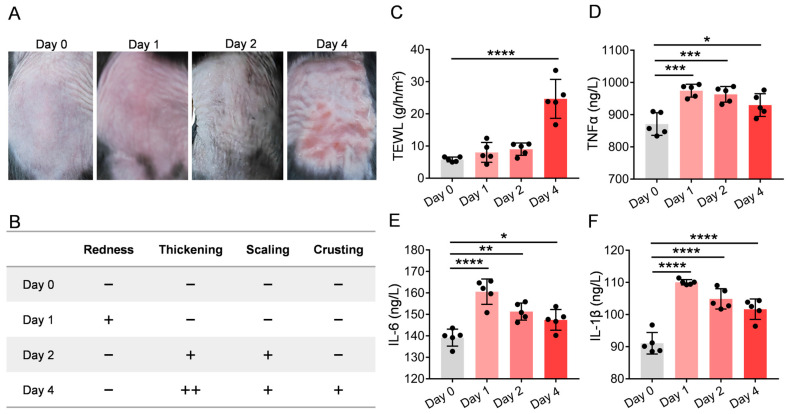
UVB irradiation impact on transepidermal water loss and inflammatory cytokine levels: Mouse skin was irradiated with a UVB dose of 300 mJ/cm^2^, and measurements were performed on days 0, 1, 2, and 4 post-irradiation. (**A**) Skin photographs at different time points; (**B**) Visual observation scores; (**C**) Transepidermal water loss (TEWL) measurements revealed significantly increased values post-irradiation compared to baseline; (**D**–**F**) UVB exposure triggered robust inflammatory responses, with markedly elevated levels of proinflammatory cytokines (TNF-α, IL-6, and IL-1β) compared to non-irradiated controls. Intergroup comparisons were performed using one-way ANOVA followed by Dunnett’s post hoc test comparing all groups to the Day 0 control group. ns, * *p* < 0.05, ** *p* < 0.01, *** *p* < 0.001, **** *p* < 0.0001. *n* = 5.

To investigate UVB’s effects on skin, we irradiated the dorsal skin of mice with 300 mJ/cm^2^ UVB and assessed skin damage at various time points post-exposure (Figure 1A). Based on the experimental observations, the skin damage on the dorsal side of the mice exhibited an aggravating trend after UVB exposure: on Day 0, all indicators (redness, thickening, scaling, and crusting) were negative (−), indicating normal skin condition; on Day 1, mild erythema (+) was observed, but no other damage was detected; on Day 2, the erythema subsided, yet mild skin thickening (+) and scaling (+) appeared; by Day 4, the damage significantly intensified, characterized by moderate skin thickening (++), persistent scaling (+), and the emergence of new crusting (+), while erythema did not reoccur. This dynamic change suggests that Day 4 post-UV exposure marks the peak period of skin damage (Figure 1B).

### 2.2. UVB Irradiation Induces Increased Transepidermal Water Loss and Release of Inflammatory Factors

Transepidermal water loss (TEWL) measurement is the most widely used objective method for evaluating skin barrier function. Increased TEWL indicates skin barrier dysfunction. We measured transepidermal water loss (TEWL) in mice skin on days 0, 1, 2, and 4 post-UV irradiation. Compared with day 0 (0.84 ± 0.75 g/m^2^/h), TEWL on day 4 post-exposure (2.68 ± 0.05 g/m^2^/h) showed a significant increase (*p* < 0.0001), indicating UV-induced disruption of the skin barrier (Figure 1C). Exposure to ultraviolet B (UVB) radiation increases the production of inflammatory cytokines, including tumor necrosis factor-α (TNF-α), interleukin-6 (IL-6), and interleukin-1β (IL-1β), which play crucial roles in inflammatory responses [15]. The levels of these inflammatory cytokines were tested on days 0, 1, 2, and 4 after irradiation using an enzyme-linked immunosorbent assay (ELISA). Compared to day 0, the expression levels of TNF-α, IL-6, and IL-1β were significantly elevated on days 1, 2, and 4 after irradiation, with the most pronounced increases observed on day 1 (Figure 1D–F).

### 2.3. UVB Irradiation-Induced Apoptosis, Neutrophil Infiltration, and Barrier Damage

**Figure 2:**
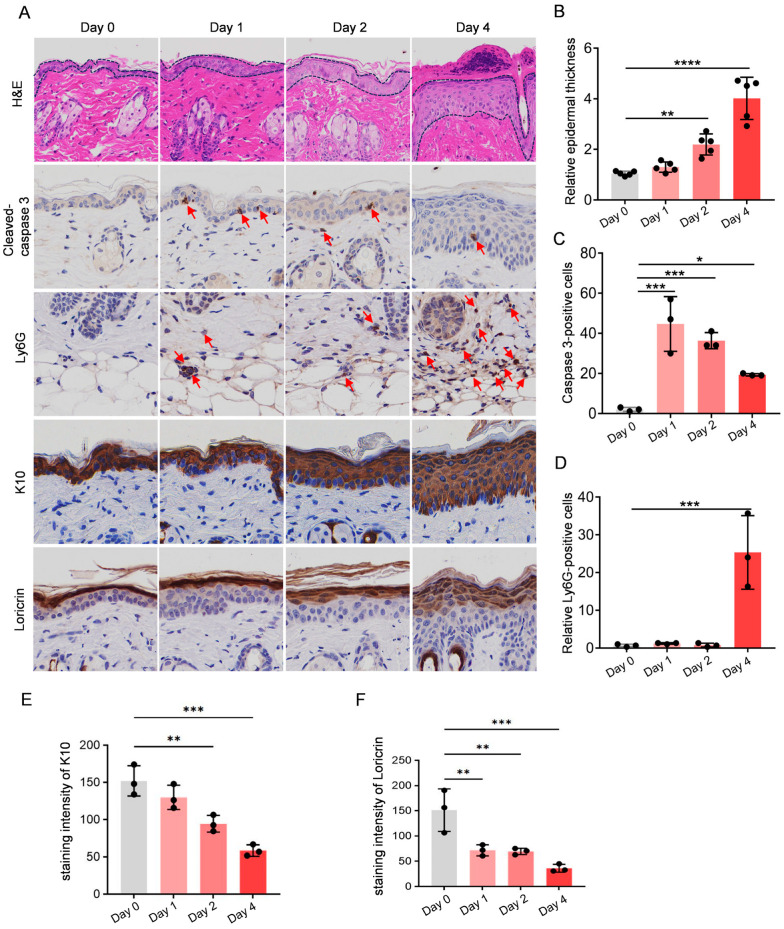
UVB irradiation induces apoptosis, facilitates neutrophil infiltration, and damages the skin barrier: (**A**) Representative H&E and immunohistochemical staining of murine skin samples collected at indicated time points post-irradiation. Scale bars: 50 μm. The dashed lines indicate significant changes in epidermal thickness, and the red arrows mark the Ly6G-positive and cleavedcaspase 3-positive cells; (**B**) Significant UV-induced epidermal thickness. *n* = 5; (**C**) Quantitative analysis of cleaved caspase-3+ cells, independent experiments; (**D**) Quantitative analysis of Ly6G; (**E**) Staining intensity of K10; (**F**) Staining intensity of Loricrin. *n* = 3. Intergroup comparisons were performed using one-way ANOVA, followed by Dunnett’s post hoc test comparing all groups to the day 0 control group. ns, * *p* < 0.05, ** *p* < 0.01, *** *p* < 0.001, **** *p* < 0.0001.

Pathological evaluation of UV-irradiated skin tissue was performed using H&E staining and immunofluorescence at different time points (days 0, 1, 2, and 4) (Figure 2A). H&E staining revealed progressive thickening of the epidermal layer over time following UV irradiation (Figure 2B). Immunohistochemistry staining was employed to label and quantify specific biomarkers, including apoptosis-associated protein (cleaved caspase 3), neutrophil marker (Ly6G), and differentiation marker (K10 and loricrin). The results demonstrated that although epidermal thickening occurred following UVB irradiation, the staining of the epidermal differentiation markers was lighter (Figure 2E,F), indicating that the epidermal barrier was compromised. Cleaved caspase 3-positive cells peaked on day 1 post-irradiation (*p* < 0.001), followed by a progressive decline (Figure 2C). Neutrophils (Ly6G), as early participants in the inflammatory response, showed a significant increase on day 4 (*p* < 0.001), indicating the induction of an inflammatory response following UV exposure (Figure 2D).

### 2.4. Scutellarin Ameliorates UVB-Induced Skin Barrier Function

**Figure 3:**
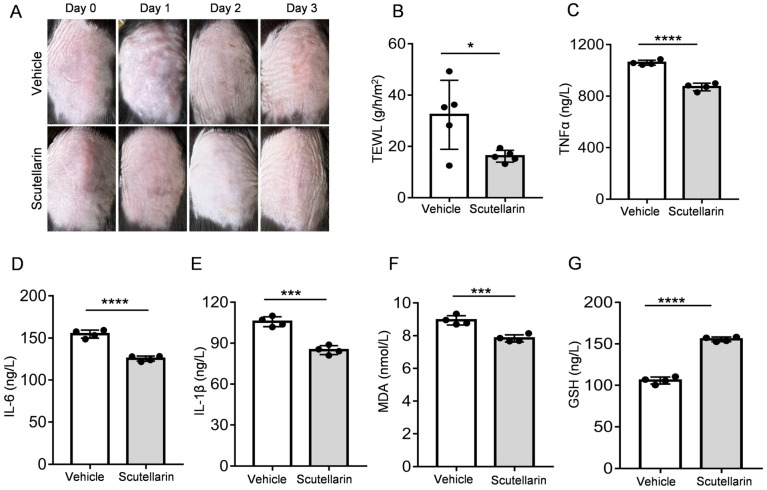
Scutellarein ameliorates TEWL and reduces cytokine production during UVB-induced skin inflammation: (**A**) Skin photographs at different time points; (**B**) TEWL (*n* = 5); (**C**–**E**) levels of cytokines based on ELISA: TNFα, IL-6, IL-1β; (**F**) levels of antioxidant capacity based on ELISA: MDA (**G**) and GSH levels. Bars represent means ± SEM, *n* = 4. * *p* < 0.05, *** *p* < 0.001, **** *p* < 0.0001 vs. the vehicle group.

To evaluate the protective effects against UV-induced skin injury, vehicle or 1% scutellarin was topically applied to the dorsal skin of mice. Macroscopic assessment of skin condition was performed through photographic documentation on days 0, 1, 2, and 3 post-irradiation. Compared with the UV-irradiated control group (vehicle), scutellarin-treated mice exhibited visibly attenuated skin damage (Figure 3A). Furthermore, transepidermal water loss (TEWL) measurements demonstrated a significant reduction in the scutellarin group (1.16 ± 2.30 g/m^2^/h) compared to the vehicle group (32.32 ± 13.44 g/m^2^/h) (*p* < 0.05; Figure 3B). These findings indicate that scutellarin mitigates UV-induced skin barrier impairment.

### 2.5. Scutellarin Attenuates UVB-Induced Inflammatory Cytokines and Oxidative Stress

Treatment with scutellarin markedly reduced cutaneous inflammation by inhibiting the release of key inflammatory mediators—tumor necrosis factor-α (TNF-α), interleukin-6 (IL-6), and interleukin-1β (IL-1β)—compared to vehicle controls (*p* < 0.001; Figure 3C–E). Concurrently, scutellarin exhibited potent antioxidant capacity by enhancing the activity of glutathione peroxidase (GPx), thereby neutralizing excess reactive oxygen species (ROS) and mitigating oxidative damage. Biochemical analyses confirmed that scutellarin significantly decreased malondialdehyde (MDA) levels (*p* < 0.001) while substantially increasing the level of reduced glutathione (GSH) activity (*p* < 0.0001) versus vehicle groups (Figure 3F,G). The results showed that scutellarin administration demonstrated significant anti-inflammatory and antioxidant effects, effectively suppressing UV-induced inflammatory cytokine production and oxidative stress.

### 2.6. Scutellarin Reduces Neutrophil Infiltration and Maintains Skin Barrier Integrity

**Figure 4:**
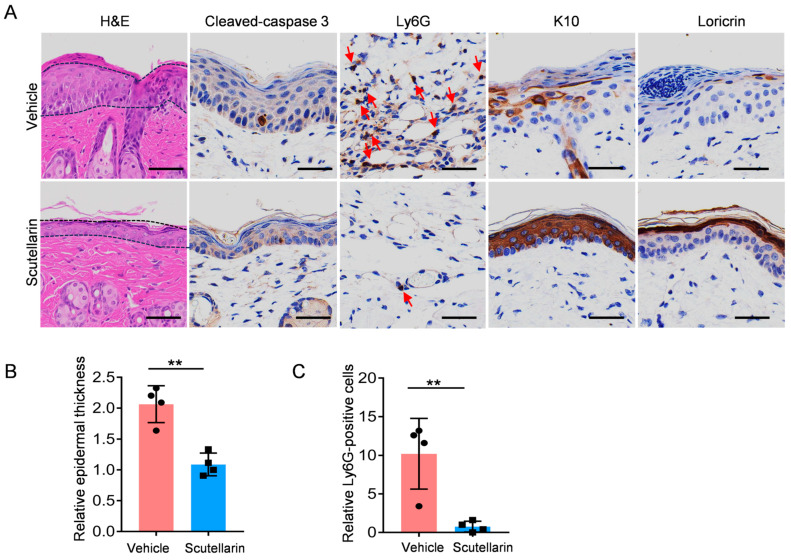
Scutellarin can reduce neutrophil infiltration and maintain barrier integrity: (**A**) Epidermal thickness was measured in samples collected 72 h post-irradiation using hematoxylin and eosin (H&E) staining, while immunohistochemical (IHC) staining was performed to evaluate cleaved caspase-3, Ly6G, K10, and loricrin. Scale bar: 50 μm. The dashed lines indicate significant changes in epidermal thickness, and the red arrows mark the Ly6G-positive cells. (**B**) Epidermal thickness quantification; (**C**) neutrophil infiltration analysis. *n* = 4. Vehicle vs. scutellarin group. ** *p* < 0.01.

This study evaluated the protective effects of scutellarein on skin damage following UVB irradiation using Hematoxylin and Eosin (H&E) staining and immunohistochemical techniques (Figure 4A). Compared to the vehicle, the scutellarein-treated group exhibited a significant reduction in epidermal thickness (*p* < 0.01; Figure 4B). Immunohistochemical staining revealed that the administration of scutellarein led to a marked decrease in the number of Ly6G positive cells. This indicates that scutellarin is capable of alleviating UVB-induced neutrophil infiltration. (*p* < 0.01; Figure 4C). Scutellarein also alleviates barrier damage and restores the expression of keratin 10 (K10) and loricrin.

### 2.7. Scutellarein Alleviates UVB-Induced Skin Damage by Modulating Inflammatory Responses and Maintaining Extracellular Matrix Homeostasis

**Figure 5:**
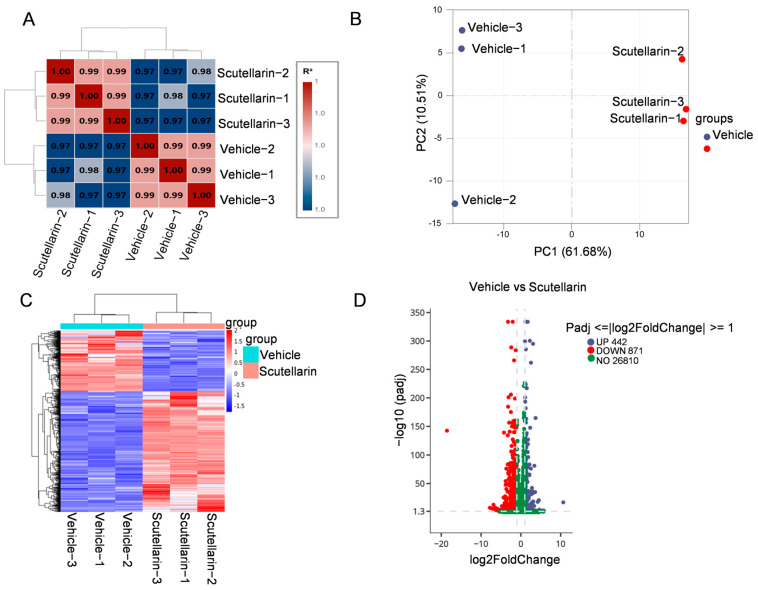
Transcriptomic profiling and differential gene expression in scutellarein vs. vehicle groups: (**A**) Inter-sample correlation heatmap. Both the x-and y-axes represent the squared correlation coefficients (R^2^) between samples; (**B**) PCA; (**C**) Clustered heatmap of differentially expressed genes (DEGs); (**D**) Volcano plot of differentially expressed genes (DEGs). The *x*-axis represents the log2-transformed fold change (log2FC) in gene expression between groups, and the *y*-axis indicates statistical significance of differential expression (−log10-transformed adjusted *p*-values, −log10padj). Upregulated genes are denoted by blue dots, while downregulated genes are marked with red dots.

**Figure 6:**
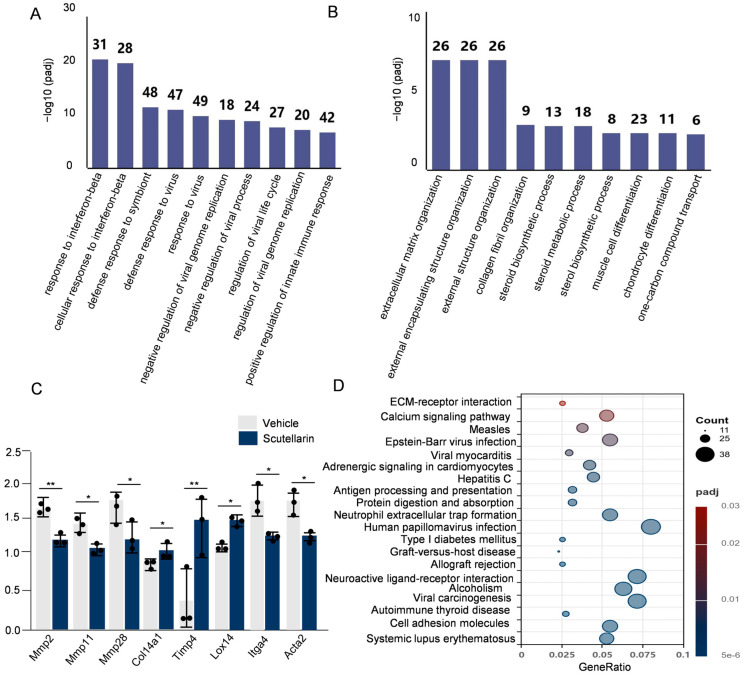
Scutellarein alleviates skin damage by modulating the expression of key regulatory genes: (**A**) GO enrichment analysis plot of upregulated genes; (**B**) GO enrichment analysis plot of downregulated genes; (**C**) Comparison of expression levels of relevant ECM genes between vehicle and scutellarin treatments; (**D**) KEGG enrichment scatter plot. Vehicle vs. scutellarin group, * *p* < 0.05, ** *p* < 0.01, *n* = 3.

To investigate the effects of scutellarein on gene expression changes in solar dermatitis, we performed transcriptome sequencing on dorsal skin tissues from different experimental groups of mice. First, we assessed the correlation of gene expression levels between samples. A correlation coefficient closer to 1 indicates higher similarity in expression patterns among samples. Based on the FPKM values of all genes in each sample, we calculated the intra-group and inter-group correlation coefficients and visualized them as a heatmap (Figure 5A). Principal component analysis (PCA) was employed to evaluate inter-group variations and intra-group sample reproducibility. The results demonstrated clear separation between the vehicle-irradiated group and scutellarein-treated group (Figure 5B). Comparative analysis of vehicle-irradiated versus scutellarein-treated groups revealed 1, 313 differentially expressed genes (DEGs) at FDR < 0.05 (log2FC > 1), comprising 442 upregulated and 871 downregulated transcripts (Figure 5D). Subsequent cluster analysis of the differentially expressed gene (DEG) set is shown in Figure 5C. We identified the 30 most significant functional terms; functional annotation revealed that in the vehicle versus scutellarein comparison, upregulated DEGs were predominantly enriched in inflammation-related processes. Downregulated DEGs showed strong associations with extracellular matrix (ECM) organization, including interferon response and collagen catabolic pathways (Figure 6A,B). Scutellarein effectively repairs UVB-induced skin damage by regulating the expression of extracellular matrix (ECM)-related genes (Figure 6C). It alleviates oxidative stress and inflammation, suppresses matrix metalloproteinase (MMP) expression, and upregulates tissue inhibitors of metalloproteinases (TIMPs), the natural inhibitors of MMPs, thereby reducing collagen degradation. Furthermore, scutellarein inhibits myofibroblast overactivation and attenuates pathological fibrosis. These synergistic actions collectively maintain ECM homeostasis and enhance skin barrier function, ultimately providing protective effects.

KEGG pathway enrichment analysis (Figure 6D) revealed significant enrichment of key biological pathways including cell adhesion molecules (CAMs), neutrophil extracellular trap formation (NETs), and ECM–receptor interactions. Cell adhesion molecules (such as ICAM1) play a critical role in intercellular recognition, adhesion, and signal transduction, mediating neutrophil infiltration into inflammatory sites and serving as key initiators of cutaneous inflammation [16]. Neutrophil extracellular trap (NET) formation represents a crucial mechanism in neutrophil-mediated immune defense. This process involves the regulation of multiple signaling pathways, such as the Mapk13-mediated signaling cascade, which influences the activation status of neutrophils and thereby modulates NET release [17]. This mechanism plays a vital role in defense against pathogen invasion. Extracellular matrix receptor interactions are crucial for maintaining cell morphology, functional performance, and tissue homeostasis. TSP-1 (Thrombospondin-1), an important component of the extracellular matrix, can bind to various cell surface receptors and participate in processes such as cell adhesion, migration, and remodeling of the extracellular matrix [18]. These findings suggest that scutellarein may exert its cutaneous protective effects by modulating inflammatory responses and restoring critical extracellular matrix homeostasis.

## 3. Discussion

This study demonstrated that scutellarein protects against and repairs UVB irradiation-induced skin damage. Based on a UVB-induced skin injury mouse model, the experiments showed that scutellarein treatment significantly reduced both cytokine secretion levels and oxidative stress responses. Meanwhile, scutellarein alleviated UVB-induced skin inflammation responses, which included reducing epidermal thickening and neutrophil infiltration. Furthermore, scutellarein attenuated UVB-induced skin injury by suppressing the aberrant overexpression of matrix metalloproteinases (MMPs) and upregulating the levels of their tissue inhibitors (TIMPs), thereby effectively maintaining the homeostasis of the extracellular matrix (ECM). The specific regulatory mechanisms involved require further investigation.

Transepidermal water loss (TEWL) assessment revealed that UVB radiation impaired skin barrier function by damaging the dermis and increasing transepidermal water loss (TEWL) [19,20]. Notably, scutellarein treatment significantly suppressed the UVB-induced elevation. This result suggests that scutellarein may effectively maintain skin barrier integrity and mitigate UVB-induced damage, potentially through mechanisms such as regulating skin lipid metabolism, enhancing stratum corneum hydration, or inhibiting the release of inflammatory mediators [21,22]. Furthermore, UVB irradiation induces acute skin inflammation, an early critical event in skin damage, characterized by skin edema primarily due to dermal vasodilation causing erythema and local tissue swelling. Histological analysis using hematoxylin and eosin (H&E) staining demonstrated that topical application of scutellarein markedly reduced the degree of skin edema and significantly attenuated epidermal thickening.

UVB irradiation activates the NF-κB and MAPK signaling pathways, promoting the release of inflammatory cytokines such as TNF-α [23,24]. Concomitantly, cytokine release induces the expression of cell adhesion molecules, including ICAM-1 and VCAM-1, on dermal endothelial cells and keratinocytes [25]. This upregulation facilitates the adhesion and migration of leukocytes, recruiting inflammatory cells (such as neutrophils) and promoting their sustained infiltration into the irradiated tissue [26]. These infiltrating inflammatory cells release enzymes, including elastases and collagenases, which contribute to skin damage and aging [27]. However, scutellarein inhibits the activation of the NF-κB signaling pathway, thereby reducing the production of these inflammatory mediators and subsequently suppressing the expression of cell adhesion molecules. Sustained UVB exposure also induces oxidative stress by triggering excessive ROS release from NADPH oxidase. Attack by ROS on lipid membranes generates substantial malondialdehyde (MDA), while non-enzymatic antioxidants, such as glutathione (GSH), are significantly depleted [28]. This manifests as elevated levels of the lipid peroxidation product MDA and reduced levels of the antioxidant molecule GSH, further exacerbating skin damage and inflammatory responses. Scutellarein directly scavenges excess ROS, mitigating oxidative damage to cell membrane lipids and reducing MDA generation. Furthermore, it modulates the intracellular antioxidant defense system by promoting GSH synthesis and enhancing cellular antioxidant capacity, thereby alleviating GSH depletion. These actions collectively protect the skin from UVB-induced oxidative damage and inflammation.

UVB irradiation induces excessive reactive oxygen species (ROS) production in skin cells, activating matrix metalloproteinases (MMPs) such as MMP-1, MMP-3, and MMP-9. These enzymes degrade collagen and extracellular matrix (ECM) components [29,30,31]. Concurrently, UVB suppresses collagen synthesis and disrupts ECM homeostasis by inducing cellular apoptosis and inflammatory responses, ultimately leading to loss of skin elasticity and increased wrinkle formation [32]. Moreover, transcriptomic analysis suggests that scutellarein inhibits both the expression and activity of MMPs, reduces collagen degradation, and restores ECM homeostasis, thereby effectively mitigating UVB-induced skin damage and photoaging.

Scutellarein demonstrates significant potential in mitigating ultraviolet (UV)-induced skin damage. It attenuates inflammatory responses by inhibiting the release of inflammatory mediators while concurrently scavenging excess reactive oxygen species (ROS), thereby reducing oxidative stress-mediated cellular injury. Additionally, scutellarein alleviates UV-induced skin edema and epidermal thickening, inhibits collagen degradation, and maintains extracellular matrix (ECM) homeostasis, effectively mitigating skin photoaging. However, inter-individual variations in skin sensitivity to UV radiation and responsiveness to scutellarein may influence therapeutic efficacy. Furthermore, potential synergistic effects with other drugs or compounds warrant further investigation. Nevertheless, scutellarein represents a promising natural therapeutic and preventive agent against UV-induced skin damage.

## 4. Materials and Methods

### 4.1. Animals

C57BL/6J mice (6-week-old, male, 18~21 g) were purchased from SPF Laboratories (Beijing Vital River, Beijing, China, certificate No. SCXK2016-0011). Six-week-old mice were shaved and chemically depilated, exhibiting unpigmented (white) skin after depilation. All animal experiments were approved by the Ethics Committee of the Beijing Institute of Radiation Medicine (approval no. IACUC-DWZX-2022-822).

### 4.2. Establishment of a Mouse Skin Damage Model Induced by UltravioletB (UVB) Irradiation

After 1–2 days of adaptation for the depilated mice, a TL 20 W/12 RS fluorescent lamp (Philips, Eindhoven, Holland) was used to irradiate the mice. The power of the lamp was 431 μW/cm^2^, detected using a UV-AB meter (Tenmars, TM-213, Taipei, Taiwan). The establishment of the UVB-induced acute skin injury mouse model was based on previous studies. Mice were exposed to a single dose of 300 mJ/cm^2^ UVB radiation. To ensure accurate and consistent dosing, the exposure time was strictly calculated based on the real-time measured intensity (0.431 mW/cm^2^) and the following formula: Time (s) = Dose (mJ/cm^2^)/Intensity (mW/cm^2^). Each mouse was irradiated for exactly 696 s. The ultraviolet intensity was measured and calibrated at a 20 cm distance before and after each irradiation session to account for any potential lamp decay. The uniformity of the radiation field was confirmed by measuring multiple points within the exposure area, and the variation was found to be less than 10%. All mice were positioned directly below the center of the light source to ensure dose homogeneity. Vertical movement of the mice was restricted by a wire mesh [33,34].

### 4.3. Macroscopic Evaluation of Dorsal Skin

The experimental mice were gently restrained and subjected to standardized dorsal skin photography at four critical time points: 0, 1, 2, and 4 days after irradiation. This schedule was designed to capture the initial response (day 1), the progression phase (day 2), and, most importantly, the peak injury plateau (day 4), which represents the stage of maximal and stable damage severity. The cutaneous reactions were evaluated through both macroscopic examination and photographic analysis with quantitative scoring of three key clinical parameters: (i) erythema intensity (scored 0–4), (ii) skin thickening (scored 0–3), (iii) scaling (scored 0–3), and (iv) crust formation (scored 0–3).

### 4.4. Measurement of Transepidermal Water Loss (TEWL)

We gently held each mouse by its limbs and pressed the probe of the measuring instrument against its dorsal skin while simultaneously pressing the measurement button to start the detection (ASCH, Osaka, Japan, VAPOSCAN AS-VT100RS). The measurement time ranged from 5 to 16 s; the shorter the measurement time, the higher the transepidermal water loss value.

### 4.5. Detection of Cytokine Levels and Oxidative Stress

Skin tissue (1.0 g) was homogenized (Torch Electromechanical Technology, Shanghai, Co., Ltd. MY03, Shanghai, China) in 9 volumes of PBS (4 °C) to obtain 10% skin tissue homogenate. Homogenates were centrifuged (3000 rpm, 20 min, 4 °C) with a microcentrifuge (Xiangyi Group, Changsha, China, TG16 W), and supernatants were carefully aspirated for downstream applications. Secreted IL-6, IL-1β, TNF-a, MDA, and GSH levels were estimated using ELISA kits (Jiangsu Meimian industrial Co., Ltd. MEIMIAN, Yancheng, China), and protein content was determined following the manufacturer’s instructions.

### 4.6. UVB Irradiation-Induced Skin Damage and the Therapeutic Effects of Scutellarein

C57BL/6J mice (6-week-old, male, 18~21 g) were randomly divided into two groups (n = 6 per group): the irradiation control group and the scutellarein treatment group. After depilating a 2 cm × 2 cm area on the dorsal skin and allowing 1–2 days for adaptation, both groups received identical UV irradiation (parameters as described previously). The irradiation control group received daily topical application of 75% ethanol (vehicle control), while the scutellarein treatment group was administered 1% scutellarein (MACKLIN, S914947, Shanghai, China) dissolved in 75% ethanol for two days prior to irradiation and for three days post-irradiation. The primary endpoint was set at 72 h post-initial UVB exposure to evaluate the protective efficacy of scutellarein while avoiding potential confounding effects from extreme tissue damage in the irradiation control group.

### 4.7. Hematoxylin and Eosin (H&E) Staining

At the end of the experiment period, mice were sacrificed via cervical dislocation, and dorsal skin specimens were harvested freshly. Skin tissues were fixed in 4% paraformaldehyde solution (Servicebio, Wuhan, China) and stained with hematoxylin and eosin (H&E). Briefly, slices were immersed in Hematoxylin staining solution (Servicebio, G1004) for approximately 1 min at room temperature to visualize cell nuclei. Then, they were rinsed thoroughly with distilled water to remove excess dye. The sections were then differentiated in Hematoxylin Differentiation Solution (Servicebio, G1039) for several seconds to remove non-specific background staining, followed by another distilled water rinse. To restore the characteristic blue coloration of the nuclei, the sections were treated with Hematoxylin Bluing Solution (Servicebio, G1040) and rinsed again with distilled water. Subsequently, the sections were dehydrated through a graded ethanol series (75%, 85%, two changes of 100%; 5 min per change), cleared in xylene (two changes; 5 min minimum per change), and finally mounted with Neutral Balsam mounting medium (China National Medicines Group Ltd. Chemical Reagents, 10004160, Shanghai, China) under glass coverslips for microscopic examination and long-term preservation. Nuclei appeared blue, while the cytoplasmic and extracellular matrix components were stained pink by the eosin step integrated within the dehydration process. We randomly selected 5–8 representative fields of view from each skin section using blind and systematic random sampling methods. We measured the epidermal thickness using ImageJ (v1.53, National Institutes of Health, Bethesda, MD, USA) and calculated the average thickness for each animal.

### 4.8. Immunohistochemistry

For immunohistochemical analysis, 4% paraformaldehyde-fixed sections were blocked with 5% bovine serum albumin and incubated with primary antibodies against Cytokeratin10 (Abcam, ab76318, Cambridgeshire, UK), loricrin (Abcam, ab198994), IL-1β (Immunoway, YT5201, San Jose, CA, USA), IL-6 (Immunoway, YT5348), C-CAS3 (Servicebio, GB11532, and LY6G (Servicebio, GB11229) overnight at 4 °C. Subsequently, tissue sections were incubated with anti-rabbit IgG (Servicebio, GB23303) at room temperature for 50 min. Subsequently, DAB (3, 3′-diaminobenzidine) chromogenic reaction was performed (Solarbio, G1212). Tissue sections were briefly immersed in hematoxylin for counterstaining and were covered with coverslips. Standardized procedures were strictly implemented, with blinding and systematic random sampling at the core. Using the threshold setting and particle analysis functions in ImageJ (v1.53, National Institutes of Health, USA), positive cells were automatically counted based on specific staining. The data were expressed as the number of positive cells per field of view, and the average number was calculated for each animal.

### 4.9. RNA-Seq Data Analysis

Total RNA was extracted from mouse dorsal skin using Trizol reagent (TIANGEN Biotech, Beijing, China), followed by mRNA enrichment and fragmentation for cDNA library construction. Quantified libraries were pooled and sequenced on the Illumina NovaSeq X Plus platform (Illumina, San Diego, CA, USA). The reference genome and corresponding gene annotation files were downloaded from the designated genomic database. HISAT2 (v2.2.1) was employed to build the genome index and align paired-end clean reads to the reference genome. Gene expression levels were quantified using featureCounts (v2.0.6) and normalized as FPKM (fragments per kilobase of transcript per million mapped reads). Differential expression analysis between groups (control-UVB and scutellarein-treated) was performed using DESeq2 (v1.42.0) in R, with significantly differentially expressed genes (DEGs) defined as those with padj < 0.05 and |log2FC| > 1. Pathway analyses were performed using GSEA software (4.0.3) and Cluster Profiler (4.4.2) software.

### 4.10. Statistical Analysis

Statistical analysis was performed using Graphpad Prism (version 9. 0.0). Results are presented as mean values ± standard error of the mean (SEM). Intergroup comparisons were performed using one way ANOVA or Dunnett’s *t*-test with *p* < 0.05 considered statistically significant.

## Data Availability

The original contributions presented in this study are included in the article. Further inquiries can be directed to the corresponding author.

## Abbreviations

The following abbreviations are used in this manuscript:

UVB	Ultraviolet B
TEWL	Transepidermal water loss
TNF-α	Tumor necrosis factor-α
IL-6	Interleukin-6
IL-1β	Interleukin-1β
MDA	Malondialdehyde
GSH	Glutathione
PCA	Principal component analysis
CAMs	Cell adhesion molecules
NETs	Neutrophil extracellular trap formation
ECM	Extracellular matrix
